# Expert Opinion on the Management of Lennox–Gastaut Syndrome: Treatment Algorithms and Practical Considerations

**DOI:** 10.3389/fneur.2017.00505

**Published:** 2017-09-29

**Authors:** J. Helen Cross, Stéphane Auvin, Mercè Falip, Pasquale Striano, Alexis Arzimanoglou

**Affiliations:** ^1^Clinical Neurosciences Section, UCL Institute of Child Health, ERN EpiCARE, London, United Kingdom; ^2^APHP, Robert Debré University Hospital, Paris, France; ^3^Epilepsy Unit, Neurology Service, Bellvitge University Hospital, L’Hospitalet de Llobregat, Barcelona, Spain; ^4^Department of Neurosciences, Rehabilitation, Ophthalmology, Genetics, Maternal and Child Health, University of Genoa, G. Gaslini Institute, Genoa, Italy; ^5^Epilepsy Unit, Child Neurology Department, Hospital San Juan de Déu, ERN EpiCARE, Barcelona, Spain; ^6^Department of Paediatric Clinical Epileptology, Sleep Disorders and Functional Neurology, ERN EpiCARE, University Hospitals of Lyon (HCL), Lyon, France

**Keywords:** algorithm, antiepileptic drug, consensus, epilepsy, epileptic and developmental encephalopathy, Lennox–Gastaut syndrome

## Abstract

Lennox–Gastaut syndrome (LGS) is a severe epileptic and developmental encephalopathy that is associated with a high rate of morbidity and mortality. It is characterized by multiple seizure types, abnormal electroencephalographic features, and intellectual disability. Although intellectual disability and associated behavioral problems are characteristic of LGS, they are not necessarily present at its outset and are therefore not part of its diagnostic criteria. LGS is typically treated with a variety of pharmacological and non-pharmacological therapies, often in combination. Management and treatment decisions can be challenging, due to the multiple seizure types and comorbidities associated with the condition. A panel of five epileptologists met to discuss consensus recommendations for LGS management, based on the latest available evidence from literature review and clinical experience. Treatment algorithms were formulated. Current evidence favors the continued use of sodium valproate (VPA) as the first-line treatment for patients with newly diagnosed *de novo* LGS. If VPA is ineffective alone, evidence supports lamotrigine, or subsequently rufinamide, as adjunctive therapy. If seizure control remains inadequate, the choice of next adjunctive antiepileptic drug (AED) should be discussed with the patient/parent/caregiver/clinical team, as current evidence is limited. Non-pharmacological therapies, including resective surgery, the ketogenic diet, vagus nerve stimulation, and callosotomy, should be considered for use alongside AED therapy from the outset of treatment. For patients with LGS that has evolved from another type of epilepsy who are already being treated with an AED other than VPA, VPA therapy should be considered if not trialed previously. Thereafter, the approach for a *de novo* patient should be followed. Where possible, no more than two AEDs should be used concomitantly. Patients with established LGS should undergo review by a neurologist specialized in epilepsy on at least an annual basis, including a thorough reassessment of their diagnosis and treatment plan. Clinicians should always be vigilant to the possibility of treatable etiologies and alert to the possibility that a patient’s diagnosis may change, since the seizure types and electroencephalographic features that characterize LGS evolve over time. To date, available treatments are unlikely to lead to seizure remission in the majority of patients and therefore the primary focus of treatment should always be optimization of learning, behavioral management, and overall quality of life.

## Overview of Lennox–Gastaut Syndrome (LGS)

Lennox–Gastaut syndrome is a severe epileptic and developmental encephalopathy, with onset typically between the ages of 3 and 7 years (most commonly 3–5 years) (1, 2). The syndrome persists through adolescence and on into adulthood, and may also, rarely, have late onset (3). LGS often occurs *de novo*, but may also evolve from other severe infantile seizure disorders, such as West syndrome (infantile spasms) (4). There is no biological marker; it has a heterogeneous etiology. Many cases occur as a result of a brain abnormality caused by, for example, a brain insult, developmental malformation, infection, or tumor (5), or a genetic mutation. However, 25–33% of cases are of unknown cause (6, 7). LGS is therefore an electroclinical entity that arises from various etiologies. Accurate diagnosis of LGS is often difficult to achieve, not only because, when taken separately, the characteristic seizure types and electroencephalogram (EEG) features are not pathognomonic, but also because they evolve and change over time. Consequently, LGS can be difficult to discern from other epilepsy syndromes, particularly early in the natural history. However, accurate and early diagnosis of the syndrome is central to its effective management, since appropriate treatment can potentially alter a patient’s clinical course and thereby improve their overall prognosis (2, 4).

### Definition of LGS

Although a precise definition of LGS has remained elusive, due to debate concerning the limits, cause(s), and diagnosis of the syndrome (4), two key criteria that were included in the original description prevail: (i) multiple seizure types, to include tonic, atonic, and atypical absence seizures, with tonic seizures predominantly occurring at night and (ii) abnormal EEG, consisting primarily of an interictal pattern of diffuse, slow spike-wave (SSW) complexes at <3Hz, occurring during wakefulness (1). Additional features, required as diagnostic by many, include paroxysmal fast rhythms (10–20 Hz) during sleep, mainly during non-rapid eye movement sleep, the hallmark of tonic seizures (8).

Although intellectual disability and associated behavioral problems were originally described in all patients by Lennox and Gastaut (1), they are not necessarily present at its outset and, consequently, their presence or absence are not included in the diagnostic criteria.

The seizure and EEG characteristics outlined above are central features of LGS; however, additional characteristics may be seen. Some or all of these may be present before or at diagnosis, or they may evolve and change over time. Additional seizure types associated with LGS include the following:

- *Epileptic spasms*: LGS develops from West syndrome in approximately 30% of pediatric cases (9, 10) and such patients may continue to have epileptic spasms during evolution to LGS (11).
- *Non-convulsive status epilepticus (NCSE)*: occurs in 50–75% of patients with LGS and usually consists of sub-continuous atypical absences with varying degrees of altered consciousness, periodically interrupted by recurring brief tonic seizures (7).
- *Focal seizures (with or without bilateral involvement), generalized tonic–clonic seizures, unilateral clonic seizures*: usually occur in the later stages of LGS, but may sometimes precede the core seizure types (4).
- *Myoclonic seizures*: occur in 11–28% of patients (6, 12–15) and can lead to falls, but are associated with many generalized epilepsies and are therefore not regarded as a defining feature of LGS (2).

### Investigations at Diagnosis

Given the aforementioned difficulties associated with accurately diagnosing LGS, several key investigations are recommended:

- *Sleep EEG*: the use of sleep EEG recording is considered mandatory for LGS diagnosis, since the occurrence of tonic seizures from sleep and/or the presence of paroxysmal fast rhythms are key diagnostic criteria, even in adult patients (4, 16). To obtain a sleep EEG in an adult patient, prior total or partial sleep deprivation may be helpful.
- *Magnetic resonance imaging (MRI)*: MRI is required to assess the presence of structural abnormalities (e.g., brain tumor/malformation, tuberous sclerosis complex) that might elucidate etiology, aid differential diagnosis, and/or guide treatment decisions (10).
- *Genetic investigation*: LGS is essentially an electroclinical diagnosis. Attempts have been made to determine a possible genetic etiology, but the genetic architecture of LGS is highly heterogeneous (17, 18). Although there is no single gene for LGS, genetic testing (i.e., targeted resequencing, array comparative genomic hybridization) can be helpful in determining an etiology and consequent recurrence risks; it may also help prevent unnecessary diagnostic investigations (19). Genetic tests that might help inform LGS etiology and diagnosis are outlined in Table 1.

**Table 1:** Examples of gene tests that might elucidate LGS genetic etiology.

Gene	Association	Reference
*SCN1A*	GEFS+/Dravet syndrome/other phenotypes	(20)
*SLC2A1*	GLUT1-deficiency syndrome	(21)
*STXBP1*	Infantile spasms/West syndrome, Lennox–Gastaut syndrome	(18)
*DNM1*	Infantile spasms/West syndrome, Lennox–Gastaut syndrome	(18)
*GABRB3*	Infantile spasms/West syndrome, Lennox–Gastaut syndrome	(18)

*GEFS+, generalized epilepsy with febrile seizures plus; GLUT1, glucose transporter type 1; LGS, Lennox–Gastaut syndrome*.

Collaboration between the EuroEPINOMICS and Epi4K/EPGP consortia allowed the analysis of exome-sequencing data from a cohort of 356 trios with LGS or infantile spasms, and found a total of 429 *de novo* mutations (17). The cohort was sufficiently large to demonstrate that this represented a statistically significant excess in comparison with the general population, supporting a prominent role for *de novo* mutations in epileptic encephalopathies. Seven genes were identified as having a role in these epileptic encephalopathies: *DNM1, ALG13, CDKL5, GABRB3, SCN1A, SCN2A*, and *STXBP1*. Overall, at least 12% of patients were found to have an identifiable causal *de novo* mutation (17).

Patients with trisomy 21 may present with late-onset LGS, characterized by frequent reflex seizures, mostly precipitated by sudden unexpected sensory stimulation (22).

### Problems with Diagnosis

Although EEG features are central to an accurate diagnosis of LGS, it is important to consider that they can take time to develop, particularly in the very young. The SSW EEG pattern may also not initially be present in patients who transition to LGS from another epilepsy syndrome, such as West syndrome (and even less in children without a history of infantile spasms). Therefore, for patients presenting with the seizure types associated with LGS, particularly tonic or atonic seizures, clinicians should be alert to the possibility of an LGS diagnosis and proactively monitor for EEG abnormalities to confirm its diagnosis. It is important to aim to identify the EEG patterns that characterize LGS early in the natural history, since they may be relatively transient: one study conducted in 64 LGS patients over a 42-year period found that the mean duration of “classic” SSW complexes was 8.2 years (range 1–35 years; median 5.5 years) (23).

None of the seizure types associated with LGS is pathognomonic and it is therefore crucial to consider a patient’s seizure pattern alongside their EEG features when an LGS diagnosis is suspected. For example, tonic or atonic drop attacks are a characteristic feature of LGS, occurring in approximately 50% of patients with SSWs, but they are not diagnostic for LGS, since they are also observed in other epilepsy syndromes, such as those with predominantly myoclonic-astatic seizures (4). The seizure types associated with LGS can also be difficult to identify. Video and sleep EEG recording may be required to detect tonic seizures from sleep together with surface electromyography recording, as stiffening may be subtle (2). Identification and quantification of atypical absence seizures can be challenging, particularly in patients whose responsiveness is impaired by intellectual disability, and where SSW is prominent in the EEG.

Other factors that can make diagnosis challenging are that the seizure types and EEG features of LGS are not static, but evolve and change over time. This is an important consideration not only for initial diagnosis but also later in a patient’s disease course, since it has implications when choosing the most appropriate treatment. The evolving nature of LGS underlines the need for regular reassessment and re-evaluation, particularly when patients are transitioning from pediatric to adult care (see Transition from Childhood to Adulthood).

### Importance of Differential Diagnosis

In most cases, careful consideration of both clinical and EEG features should allow LGS to be discernible from other epilepsy syndromes; however, there are certain epilepsy syndromes with which it may be confused (2). Although myoclonic-atonic epilepsy (MAE; Doose syndrome) has specific diagnostic criteria (24), at diagnosis the recurrent myoclonic atonic seizures seen as drop attacks may lead to a presumptive diagnosis of LGS. This particular example illustrates where delineating a correct syndrome diagnosis is of considerable prognostic importance; approximately two-thirds of patients with MAE have a favorable prognosis (25), whereas in LGS this is lacking. Atypical benign partial epilepsy of childhood can be confused with LGS because it has somewhat similar EEG features, but these differ from those of LGS in having normal or slightly slow background activity and in lacking fast spike discharges (>10/s), with an absence of tonic seizures (2). The syndrome of Epileptic Encephalopathy with Continuous Spike Wave of Slow Sleep may also manifest with drop attacks as a predominant seizure manifestation, with evidence of neurodevelopmental regression at presentation, but on evaluation although bursts of SSW are seen with atonic absence seizures when awake, prolonged sleep recordings have failed to disclose tonic seizures (26). Some forms of focal epilepsy, such as those involving the supplementary sensorimotor area, where tonic, atonic, and drop attacks may manifest, may also be misdiagnosed. Seizures arising from the supplementary sensorimotor area result in a set of clinical phenomena that usually include brief asymmetric tonic posturing of the extremities with preserved consciousness (27, 28). EEG with additional surface electromyography electrodes can be useful in differentiating symmetric or asymmetric tonic seizures. Moreover, focal frontal or parietal epilepsies can be differentiated from LGS as the interictal EEG usually shows normal background activity, the ictal EEG in tonic asymmetric seizures can be normal, and fast spike discharges are either localized over the vertex or diffuse but clearly asymmetric (29).

In around 50% of West syndrome cases, the emergence LGS is later seen (30). The progressive evolution of features of LGS, and the fact that it may precede or follow other epilepsy syndromes and/or share overlapping features with them, has led to the hypothesis that it may be a developmental form of epilepsy with a continuum from other forms of epilepsy and ultimately LGS (31–33). This further underlines the need for continual reappraisal and re-evaluation of diagnoses, so that treatment can be tailored appropriately.

## Review of Current Evidence for Treatments for LGS

### Pharmacological Treatment of LGS

The evidence base for pharmacological treatment of LGS is limited. Epidemiological follow-up data and review of adults with LGS suggest that, with standard treatments, there is a very low likelihood of remission in the long term, confirming the ongoing challenges faced in seizure management (16, 34). It should also be noted that spontaneous fluctuations in the frequency and type of seizures are likely to occur (35). Consequently, when considering treatment response, the duration of time over which this is assessed also needs to be taken into account.

A Cochrane review of antiepileptic drug (AED) treatment for LGS, conducted in 2013, included eight studies of seven drugs utilized as adjunctive therapy (36). Meta-analysis was not possible due to differences in study designs, inclusion/exclusion criteria, and outcomes. The conclusion was that no one drug appears highly effective in LGS over and above another: lamotrigine (LTG), rufinamide (RUF), topiramate (TPM), and felbamate (FLB) may all be useful as adjunctive therapy and clobazam (CLB) may be useful for drop attacks. No benefit was shown for cinromide or low-/high-dose thyrotropin-releasing hormone (36). A recent search of the literature has not revealed any additional randomized controlled trials (RCTs) that would add to this analysis to date.

#### Antiepileptic Drugs Licensed or Widely Used for LGS in Europe and the USA

##### Sodium Valproate (VPA)

Valproate has never been specifically licensed for use in LGS (Table 2). However, most patients presenting with epilepsy characterized by generalized or multiple seizure types will initially be prescribed VPA in view of the fact that it is broad spectrum and highly unlikely to lead to aggravation of seizures. Consequently, it is likely to have been utilized first line in children presenting with *de novo* or emerging LGS. A major consideration in the general use of VPA has been the associated teratogenic and developmental risk to an unborn child of a mother taking VPA (37–39). International League Against Epilepsy guidelines recommend that VPA should be avoided where possible in women of childbearing potential, but recognize that, for seizure (or epilepsy) types where VPA is the most effective treatment, the risks and benefits of VPA and other treatment alternatives should be discussed, and that VPA should be offered as a first-line treatment for epilepsy syndromes where it is the most effective treatment (40). In line with these recommendations, reviewing risk–benefit, conception in many with LGS is not a consideration and consequently the benefit of utilization of VPA often outweighs any risk.

**Table 2:** Summary of AEDs licensed or widely used for LGS in Europe and/or the USA.

AED	Phase III efficacy in drop attacks	Phase III efficacy in other seizures associated with LGS	Effect on cognition	Behavioral AEs	Other considerations
VPA	No Phase III study in LGS	No Phase III study in LGS	Common^a^ AEs: confusional state, disturbance in attention, stupor, memory impairment^j^ Rare^b^ AEs: cognitive disorder, learning disorder^j^ An increase in alertness may occur^j^	Common^a^ AEs: aggression, agitation^j^ Rare^b^ AE: abnormal behavior^j^ Aggression, hyperactivity, and behavioral deterioration have occasionally been reported^j^	Should not be used as first-line treatment in female adolescents, in women of childbearing potential and pregnant women unless alternative treatments are ineffective or not tolerated because of high teratogenic potential and risk of developmental disorders in infants exposed *in utero*^j^

LTG	Phase III placebo-controlled RCT in LGS (16-week maintenance period) (41): Significantly greater reduction in drop attacks^c^ for LTG vs. PBO (−34 vs. −9%; *p* = 0.01) Significantly higher drop attack responder rate^d^ for LTG vs. PBO (37 vs. 22%; *p* = 0.04) Freedom from drop attacks not reported	Phase III placebo-controlled RCT in LGS (16-week maintenance period) (41): Significantly greater reduction in all seizures for LTG vs. PBO (−32 vs. −9%; *p* = 0.002) Significantly greater reduction in tonic–clonic seizures for LTG vs. PBO (−36 vs. +10%; *p* = 0.03) Significantly higher all seizure responder rate^d^ for LTG vs. PBO (33 vs. 16%; *p* = 0.01) Significantly higher tonic–atonic seizure responder rate^d^ for LTG vs. PBO (43 vs. 20%; *p* = 0.007) Seizure freedom not reported	Very rare^e^ AEs: confusion, hallucinations^k^	Common^a^ AEs: aggression, irritability, agitation^k^	VPA inhibits LTG glucuronidation, reducing its metabolism and increasing its half-life nearly twofold; therefore, lower LTG doses required when used concomitantly with VPA^k^ Dose increase required following withdrawal of concomitant VPA therapy

RUF	Phase III placebo-controlled RCT in LGS (12-week treatment period) (42): Significantly greater reduction in drop attacks^f^ for RUF vs. PBO (−42.5 vs. +1.4%; *p* < 0.0001) Significantly higher drop attack responder rate^d^ for RUF vs. PBO (42.5 vs. 16.7%; *p* = 0.002) Freedom from drop attacks^f^ occurred in 4.1% RUF patients vs. 3.3% PBO patients (*p* = NS)	Phase III placebo-controlled RCT in LGS (12-week treatment period) (42): Significantly greater reduction in all seizures for RUF vs. PBO (−32.7 vs. −11.7%; *p* = 0.0015) Significantly higher all seizure responder rate^d^ for RUF vs. PBO (31.1 vs. 10.9%; *p* = 0.0045) Significantly greater reduction in absence and atypical absence seizures for RUF vs. PBO (−50.6 vs. −29.8%; *p* = 0.0222) Significantly greater reduction in atonic seizures for RUF vs. PBO (−44.8 vs. −21.0%; *p* = 0.0125) No patients were seizure-free during the study Significantly higher percentage of RUF vs. PBO patients experienced an improvement in seizure severity on parental/guardian global evaluation scale (53.4 vs. 30.6%; *p* = 0.0041)		Common^a^ AE: anxiety^l^	Common^a^ AEs: weight decrease, anorexia, eating disorder, decreased appetite^l^

TPM	Phase III placebo-controlled RCT in LGS (11-week double-blind treatment period) (43): Significantly greater reduction in drop attacks^f^ for TPM vs. PBO (−14.8 vs. +5.1%; *p* = 0.041)	Phase III placebo-controlled RCT in LGS (11-week double-blind treatment period) (43): Significantly greater reduction in major seizures^g^ for TPM vs. PBO (−25.8 vs. +5.2%; *p* = 0.015)	Common^a^ AEs: bradyphrenia, expressive language disorder, confusional state, disorientation, disturbance in attention, memory impairment, amnesia, cognitive disorder, mental impairment, psychomotor skills impaired^m^	Very common^i^ AE: depression^m^	Rare AE: Stevens–Johnson syndrome^m^
	Higher (but not significantly) drop attack responder rate^d^ for TPM vs. PBO (28 vs. 14%; *p* = 0.071) During double-blind phase, 2.2% TPM vs. 0% PBO patients were free from drop attacks^f^ During maintenance period (last 8 weeks of double-blind phase), 10.9% TPM vs. 0% PBO patients were free from drop attacks^f^	Significantly higher major seizure^g^ responder rate^d^ for TPM vs. PBO (33 vs. 8%; *p* = 0.002) During double-blind phase, 2.2 TPM vs. 0% PBO patients were free from major seizures^g^ Significantly more TPM vs. PBO patients experienced improvement in seizure severity on parental global evaluation scale (*p* = 0.037)	Many uncommon^h^ cognitive AEs^m^	Common^a^ AEs: aggression, mood altered, agitation, mood swings, depressed mood, anger, abnormal behavior^m^ Many uncommon^h^ behavioral AEs^m^

CLB	Phase III placebo-controlled RCT in LGS (12-week maintenance period) (44): Significantly greater reduction in drop attacks for CLB vs. PBO: average weekly rates decreased 12.1% for PBO vs. 41.2% (*p* = 0.0120), 49.4% (*p* = 0.0015) and 68.3% (*p* < 0.0001) for CLB 0.25, 0.5, and 1 mg/kg/day, respectively Significantly higher drop attack responder rate^d^ for CLB vs. PBO: 31.6% for PBO vs. 43.4% (*p* = NS), 58.6% (*p* = 0.0159), and 77.6% (*p* < 0.0001) for CLB 0.25, 0.5, and 1.0 mg/kg/day, respectively Rate of freedom from drop attacks was 3.5% for PBO vs. 7.5, 12.1, and 24.5% for CLB 0.25, 0.5, and 1 mg/kg/day, respectively (statistical comparison not valid due to low patient numbers)	Phase III placebo-controlled RCT in LGS (12-week maintenance period) (44): Significantly greater reduction in total seizures for CLB vs. PBO: average weekly rates decreased 9.3% for PBO vs. 34.8% (*p* = 0.0414), 45.3% (*p* = 0.0044) and 65.3% (*p* < 0.0001) for CLB 0.25, 0.5, and 1 mg/kg/day, respectively	AEs include: slowing of reaction time, confusion, slowed or indistinct speech^n^ Anterograde amnesia may occur, especially at higher doses^n^	AEs include: numbed emotions, restlessness, irritability, acute agitational states, anxiety, aggressiveness, delusion, fits of rage, nightmares, hallucinations, psychotic reactions, suicidal tendencies^n^ Amnesia effects may be associated with inappropriate behavior^n^	Tolerance and physical and/or psychic dependence may develop, especially during prolonged use^n^: in a long-term, single-center LGS study, tolerance developed in 48% of initial responders (45) Discontinuation may result in withdrawal or rebound phenomena^n^ Therapeutic benefit must be balanced against the risk of habituation and dependence during prolonged use^n^ May change VPA plasma levels when used concomitantly; plasma levels of VPA should therefore be monitored^n^ May decrease LTG plasma levels (expert opinion)

FLB	Placebo-controlled RCT in LGS (70-day treatment period; 56-day maintenance period) (46): During treatment period, significantly greater reduction in atonic seizures for FLB vs. PBO (−34 vs. −9%; *p* = 0.01); 3/28 FLB-treated patients vs. 0/22 PBO-treated patients had no atonic seizures	Placebo-controlled RCT in LGS (70-day treatment period; 56-day maintenance period) (46): During treatment period, significantly greater reduction in parental counts of all seizures for FLB vs. PBO (−19 vs. +4%; *p* = 0.002); no patients were seizure-free During maintenance period, significantly greater reduction in parental counts of all seizures for FLB vs. PBO (−26 vs. +5%; *p* < 0.001); 4/37 FLB-treated patients vs. 1/36 PBO-treated patients had no seizures	In controlled pediatric LGS studies, 6.5% of patients reported abnormal thinking^o^	Frequent AEs: agitation, psychological disturbance, aggressive reaction^o^ Infrequent AEs: hallucination, euphoria, suicide attempt^o^	Black box warning over risk of aplastic anemia and acute liver failure^o^
	During maintenance period, significantly greater reduction in atonic seizures for FLB vs. PBO (−44 vs. −7%; *p* = 0.002); 5/28 FLB-treated patients vs. 0/22 PBO-treated patients had no atonic seizures	During treatment period, reduction in generalized tonic–clonic seizures was −28% for FLB vs. +11% for PBO (*p* = NS); 2/16 FLB-treated patients vs. 1/13 PBO-treated patients had no generalized tonic–clonic seizures During maintenance period, significantly greater reduction in generalized tonic–clonic seizures for FLB vs. PBO (−40 vs. +12%; *p* = 0.017); 7/16 FLB-treated patients vs. 1/13 PBO-treated patients had no generalized tonic–clonic seizures During maintenance period, significantly higher parent/guardian global evaluation scores for FLB vs. PBO from day 49 onward (*p* < 0.001)		In controlled pediatric LGS studies, 16.1% of patients reported nervousness and 6.5% of patients reported emotional lability^o^	Only available for limited use in Europe on a patient-by-patient basis, due to risk of aplastic anemia and liver failure

*AE, adverse event; AED, antiepileptic drug; CLB, clobazam; FLB, felbamate; LGS, Lennox–Gastaut syndrome; LTG, lamotrigine; NS, not significant; PBO, placebo; RCT, randomized controlled trial; RUF, rufinamide; TPM, topiramate; VPA, valproate*.

*^a^≥1/100 to <1/10*.

*^b^≥1/10,000 to <1/1,000*.

*^c^Tonic, atonic, and major myoclonic seizures*.

*^d^≥50% seizure frequency reduction from baseline*.

*^e^<1/10,000*.

*^f^Tonic–atonic seizures*.

*^g^Drop attacks and tonic–clonic seizures*.

*^h^≥1/1,000 to <1/100*.

*^i^≥1/10*.

*^j^Epilim (sodium valproate) Summary of Product Characteristics: https://www.medicines.org.uk/emc/medicine/6781*.

*^k^Lamictal (lamotrigine) Summary of Product Characteristics: http://www.ema.europa.eu/docs/en_GB/document_library/Referrals_document/Lamictal_30/WC500008824.pdf*.

*^l^Inovelon (rufinamide) Summary of Product Characteristics: http://www.ema.europa.eu/docs/en_GB/document_library/EPAR_-_Product_Information/human/000660/WC500032937.pdf*.

*^m^Topamax (topiramate) Summary of Product Characteristics: http://www.ema.europa.eu/docs/en_GB/document_library/Referrals_document/Topamax_30/WC500018620.pdf*.

*^n^Frisium (clobazam) Summary of Product Characteristics: http://www.medicines.org.uk/emc/medicine/8298/SPC/Frisium+Tablets+10+mg*.

*^o^Felbatol (felbamate) Prescribing Information: http://www.accessdata.fda.gov/drugsatfda_docs/label/2009/020189s022lbl.pdf*.

##### Lamotrigine

Lamotrigine is licensed in Europe^1^ and the USA^2^ for the treatment of seizures associated with LGS. LTG was shown to be effective and well tolerated in the treatment of LGS in a Phase III placebo-controlled RCT conducted in 169 patients with LGS (41) (Table 2). In addition, in a double-blind, placebo-controlled, crossover study conducted in 30 pediatric and adolescent patients with refractory generalized epilepsy, 20 of whom had LGS, LTG was well tolerated and a statistically significant reduction in seizure frequency was observed with LTG, compared with placebo (47). VPA inhibits the metabolism of LTG (48). Therefore, a decreased LTG dose is required when used as add-on to VPA therapy (see text footnote 1). Conversely, if VPA is subsequently discontinued, the LTG dose may need to be increased. Monitoring and/or dose adjustment of these AEDs when used in combination is recommended, particularly as many experts feel that the combination of LTG with VPA is one of the best for the early treatment of LGS (49).

##### Rufinamide

Rufinamide is licensed in Europe^3^ and the USA^4^ as adjunctive treatment for seizures associated with LGS. RUF’s effectiveness in LGS was demonstrated in a Phase III placebo-controlled RCT conducted in 138 patients with LGS (42) (Table 2). This RCT was followed by a long-term, open-label extension study, in which all patients (*n* = 124) received RUF for a median of 432 days (50). Reductions in seizure frequency were maintained throughout the study and tolerability observed in the initial trial was also maintained with long-term treatment (50). A *post hoc* subgroup analysis of data from the 31 adult patients (age 18–37 years) included in the initial Phase III trial demonstrated that RUF had favorable efficacy and was generally well tolerated when used as adjunctive treatment for adults (51). Responder rates were 33.3% for RUF versus 0% for placebo (*p* = 0.066) for all seizures, and 57.1 versus 10.0% (*p* = 0.020) for drop attacks (51). The long-term safety and seizure outcome of adjunctive RUF therapy was recently evaluated in Japanese LGS patients in an open-label extension study following a 12-week multicenter, placebo-controlled RCT (52). For the 41 patients who completed the study, the median percent change in the frequency of tonic–atonic seizures relative to the frequency at the start of the double-blind study was −39.3% (12 weeks), −40.6% (24 weeks), −46.8% (32 weeks), −47.6% (40 weeks), and −36.1% (52 weeks). Reduction of total seizure frequency was also maintained until 52 weeks. Adverse events (AEs) were mild or moderate, except for transient seizure aggravation in three patients. AEs resulting in discontinuation of RUF were decreased appetite, drug eruption, and worsening of underlying autism (52).

##### Topiramate

Topiramate is licensed in Europe^5^ and the USA^6^ as adjunctive therapy for the treatment of seizures associated with LGS. TPM was shown to be effective in the treatment of LGS in a Phase III placebo-controlled RCT conducted in 98 patients with LGS (43) (Table 2). Ninety-seven patients completing the RCT continued into an open-label extension study with flexible dosing (53). For patients who completed 6 months of treatment (*n* = 84), the drop attack responder rate (response defined as ≥50% reduction from RCT baseline) was 55 and 15% of patients had no drop attacks for ≥6 months (53). TPM was well tolerated and 71% of patients were retained on therapy for ≥3 years. Overall, 5% of patients reported behavioral problems (53). TPM has a high association with cognitive and behavioral AEs and may, rarely, cause Stevens–Johnson syndrome (see text footnote 5).

##### Clobazam

Clobazam is licensed in Europe^7^ as adjunctive therapy in epilepsy, and in the USA^8^ as adjunctive treatment for seizures associated with LGS. The effectiveness of CLB in the treatment of LGS was demonstrated in a placebo-controlled Phase III RCT conducted in 238 patients with LGS (44) (Table 2). Patients completing this RCT and a previous Phase II study (54) were eligible to enter a long-term, open-label extension study (*n* = 267) (55). Seizure frequency reduction was maintained over the long term: median reductions from baseline in drop attacks and total seizures were −92% (*n* = 113) and −82% (*n* = 118), respectively, after 3 years, and −91% (*n* = 42) and −85% (*n* = 43), respectively, after 5 years (55). At Years 1 and 3, 14% of patients who were initial responders (defined as ≥50% seizure reduction from baseline to Month 3) had lost their responses, potentially indicating tolerance (55).

Important considerations in the use of CLB are that it is associated with a range of cognitive and behavioral AEs as well as a reported high risk of tolerance. The therapeutic benefit of CLB must therefore be balanced against the risk of habituation and dependence during prolonged use. Studies suggest, however, that only approximately one-third of patients develop tolerance (56, 57). CLB can be useful in the treatment of “difficult phases”/“crisis episodes” of LGS, such as the occurrence of cluster seizures, prolonged absences, and NCSE. Consequently, due to the risk of tolerance, CLB should generally only initially be considered for use on an intermittent basis over a period of 3–5 days (expert opinion). That aside, particularly when drop attacks are problematic, CLB can be considered for regular use with awareness of the possibility of habituation. When used acutely, CLB does not appear to be associated with exacerbation of tonic seizures or status epilepticus, unlike some other benzodiazepines (expert opinion).

##### Felbamate

In view of an early suggestion of a high risk of aplastic anemia and acute liver failure, FLB has not been approved for use by the European Medicines Agency (EMA). Nevertheless, it can be an extremely useful medication and is approved for limited use in some European countries, on a patient-by-patient basis (58). The effectiveness of adjunctive FLB treatment in LGS was demonstrated in a placebo-controlled RCT (46) (Table 2). Patients completing this trial could continue into a 12-month, open-label extension study, during which improvement in seizure control observed in the initial trial was sustained: at Month 12, responder rates (response defined as >50% reduction from RCT baseline) were approximately 50% for total seizures and approximately 67% for astatic seizures (59). In the USA, FLB carries a black box warning and is licensed as monotherapy or adjunctive therapy for treatment of seizures associated with LGS only in patients who respond inadequately to other treatments and whose epilepsy is so severe that a risk of aplastic anemia and/or liver failure is deemed acceptable in light of the benefits conferred by its use.^9^ Due to its known risks and associated licensing restriction, FLB has a limited role in the management of LGS.

#### Use of Other Pharmacological Agents in the Treatment of LGS

Cannabidiol has some evidence of efficacy and an adequate safety profile when used as adjunctive therapy in children and young adults with treatment-resistant epilepsies, including LGS and Dravet syndrome (60, 61). It is currently being assessed as a potential adjunctive treatment for children and adults with LGS in Phase III clinical trials (ClinicalTrials.gov^10^ identifiers: NCT02318602, NCT02224690, NCT02224560, and NCT02318537). In the first of these trials to report results,^11^ adjunctive cannabidiol was shown to significantly reduce the monthly frequency of drop attacks over a 14-week treatment period (primary endpoint), compared with placebo, in patients aged 2–55 years with a confirmed diagnosis of drug-resistant LGS (*p* = 0.0135). Cannabidiol was generally well tolerated and the most common AEs (>10% of cannabidiol-treated patients) were diarrhea, somnolence, decreased appetite, pyrexia, and vomiting (see text footnote 11). Some of these side effects may be attributable to comedication; cannabidiol is a potent inhibitor of enzymes in the cytochrome P450 pathway, and a definitive interaction has been reported with at least CLB (62).

Despite having scarce evidence of effectiveness specifically in LGS, some broad-spectrum AEDs, including levetiracetam (LEV), zonisamide (ZNS), and perampanel (PER), may be useful. An open-label, multicenter, observational clinical study assessed the efficacy and tolerability of adjunctive LEV in 55 patients with LGS over an 8-week maintenance period (63). Thirty-two (58.2%) patients experienced >50% seizure frequency reduction and 15 (27.3%) became seizure free; a > 50% seizure frequency reduction was observed in seven of 12 (58.3%) patients with drop attacks. The most common AE was hyperactivity (12.7%) (63). A Korean multicenter study assessed the efficacy and safety of adjunctive ZNS in 62 patients with LGS in whom therapy was maintained for ≥12 months (64). Overall, 32 (51.6%) patients experienced >50% seizure frequency reduction, of whom three achieved seizure freedom. AEs included transient somnolence and anorexia (64). A multicenter, observational, retrospective survey was conducted of 58 children and adolescents with various refractory epilepsies, including five with LGS, who were treated with adjunctive PER (65). After the first 3 months of therapy, the responder rate (≥50% seizure frequency reduction) for all included patients was 31% (18/58) overall and five patients (9.1%) achieved seizure freedom. Seizure aggravation was observed in five (9.1%) patients and the most frequently reported AEs were reduced vigilance or fatigue (*n* = 16) and behavioral changes (*n* = 14) (65).

Steroids may occasionally be helpful for controlling atypical absence seizures, drop attacks, and NCSE, but relapse is common. No RCT of steroids in LGS has yet been conducted (4). In addition, prolonged use of steroids is associated with serious AEs, including growth suppression, hyperlipidemia, and osteoporosis (66). It is recommended that steroids only be used for “crisis” periods with a clear plan for short-term use and wean, avoiding long-term use.

### Non-Pharmacological Treatment of LGS

#### Ketogenic Diet (KD) Therapy

The KD is a high-fat diet, designed to mimic the metabolic effects of starvation, which can be administered in a variety of ways (e.g., classical KD, medium chain triglyceride KD, modified KD). In a placebo-controlled RCT conducted in 145 children with drug-resistant epilepsies, the responder rate (>50% seizure frequency reduction) after 3 months was significantly higher for the KD versus no change in treatment (38 vs. 6%; *p* < 0.0001) (67). The most common AEs were constipation, vomiting, hunger, and lack of energy (67). In a further RCT, conducted only in children with LGS, children were fasted and subsequently had seizure counts and EEG (68). They were then all commenced on the KD, but were randomized to receive a glucose or saccharin drink, the former expecting to alleviate the ketosis. This was a crossover trial, so, at 6 days, each were again fasted and then initiated on the KD with the contrary drink. At the end of the study, the difference in seizure counts between the two groups did not quite reach significance. There were, however, methodological flaws to the study; the glucose group never lost their ketosis, and seizure counts were initiated after fasting (68). Several open-label studies have investigated the effectiveness of KD therapy specifically in patients with LGS. In a single-center retrospective review of 71 patients with LGS treated with the KD, the responder rate (>50% seizure frequency reduction) after 6 months was 51%; 23% of patients experienced >90% seizure reduction and one patient (1%) achieved seizure freedom (intention-to-treat analysis) (69). Similar results were observed after 12 months (69). In a further retrospective study of 47 patients with LGS treated with KD therapy at a single center in China, responder rates (≥50% seizure frequency reduction) after 3 and 6 months were 49 and 53%, respectively, and 10% of patients were seizure free after 6 months (70). Early improvement in the EEG background pattern and a reduction in interictal epileptic discharges were associated with significant seizure reduction, compared with the absence of such EEG changes (*p* < 0.01) (70). In a single-center prospective study, 20/61 patients with LGS were treated with KD therapy (71). After 18 months, 15 (75%) patients remained on the diet, 8 (40%) were responders (≥50% seizure frequency reduction), and 3 (15%) were seizure free (71). In another single-center retrospective review of 25 children with LGS treated with the modified Atkins diet (in which carbohydrate intake was restricted to 10 g/day), 2 (8%) patients were seizure free, and 12 (48%) were responders (>50% seizure frequency) after 3 months (72). After 6 months, 11 (44%) patients remained on the diet, 3 (12%) were seizure free, and all 11 (44%) were responders. After 1 year, 9 (36%) patients remained on the diet, 3 (12%) were seizure free, and all 9 (36%) were responders (72). A literature review of 18 studies that included outcome data specifically for LGS patients found that 88/189 (47%) children experienced >50% seizure frequency reduction after 3–36 months of KD therapy (69).

As with pharmacological treatment options, the effectiveness of KD therapy is dependent on patient adherence, which may be affected by an individual’s food preferences, particularly in the presence of coexistent rigid behavior (e.g., associated with autism). Its use therefore requires a dedicated KD team and committed carers. Studies conducted in LGS patients generally did not report difficulties in administering the diet (69–72). Although reported AEs with KD therapy may be relatively common, most can generally be alleviated by making adjustments to the diet, and successful implementation may allow AED treatment(s) to be tapered and discontinued. Another advantage of the KD is that, if a response is to occur, it is usually observed within 3 months, allowing the effectiveness of this treatment option in an individual patient to be assessed relatively quickly (expert opinion). However, consideration has to be given to duration of treatment in responders, and the risk–benefit of long-term utilization taken into consideration at all times (73).

#### Resective Surgery

Lennox–Gastaut syndrome is an electroclinical syndrome, which may have focal pathology as etiology. Successful surgical outcomes have been reported in patients with a congenital brain abnormality that was predominantly focal on MRI, despite most of the epileptiform discharges being generalized (74). It is mandatory to look for a potential localized brain abnormality in patients with LGS, although this is not frequently found. Early-onset epilepsy, associated with a localized brain lesion, can evolve to an electroclinical picture mimicking LGS, when resective surgery may be curative. Consequently, a high index of suspicion should be utilized when assessing particularly atypical presentations with high-quality neuroimaging. When no lesion is identified, patients can be treated with palliative surgery [vagus nerve stimulation (VNS) or corpus callosotomy—see Vagus Nerve Stimulation and Corpus Callosotomy sections]. After corpus callosotomy or VNS therapy, generalized epileptiform discharges may be more localized and it may therefore be appropriate to re-evaluate the patient for the presence of a seizure focus following these procedures (75). However, in the absence of a definitive structural lesion, resective surgery is unlikely to be of benefit.

#### Vagus Nerve Stimulation

The use of VNS in drug-resistant epilepsy is well established (76), with some evidence of effectiveness in LGS (76–81). A six-center, retrospective study evaluated the effectiveness of VNS therapy in 50 patients with LGS, by comparing pre- and postimplantation seizure frequency (79). Median reductions in total seizures were 42% at 1 month, 58.2% at 3 months, and 57.9% at 6 months postimplantation (*p* < 0.0001 at each timepoint). Similarly, median reductions in drop attacks were 47% at 1 month (*p* < 0.0001), 55% at 3 months (*p* < 0001), and 88% after 6 months (*p* = 0.0002), and median reductions in atypical absence seizures were 48, 73, and 81% after 1, 3, and 6 months, respectively (statistical significance not stated). In five patients who had previously undergone corpus callosotomy, reduction in total seizures was 73% after 3 months and 69% after 6 months (79). In a single-center retrospective study conducted in Korea, 10 LGS patients who underwent VNS were followed up for 24 months (80). Seven (70.0%) patients were responders (>50% seizure frequency reduction) and two (20.0%) experienced >75% seizure frequency reduction. These results were similar to those observed for 14 LGS patients who underwent corpus callosotomy at the same center (80). A pan-European, retrospective, multicenter study investigated the effectiveness of VNS in 347 children with drug-resistant epilepsy over a follow-up period of up to 24 months (81). In patients with LGS, responder rates at 6, 12, and 24 months after implantation were 28.5% (35/123), 32.9% (48/146), and 39.1% (34/87), respectively (81). These responder rates in LGS are consistent with responder rates for VNS in other types of epilepsy.

An evidence-based guideline published by the American Academy of Neurology included a pooled analysis of 113 patients with LGS treated with VNS in four studies, demonstrating a responder rate of 55% (77). On the basis of this evidence, the academy recommended that VNS may be considered in patients with LGS (Level C) (77). VNS does not interact with AEDs. AEs are mainly stimulation related, mild-to-moderate in severity, reversible, and tend to decrease over time, seldom necessitating removal of the device (82). Patients treated with VNS appear to show continuing improvement over time and some demonstrate improvement in alertness (83). Although VNS is less invasive than callosotomy, it involves the implantation of a device, to which some patients and/or parents/guardians may be averse. An advantage of VNS is that it can be used in conjunction with other forms of pharmacological and non-pharmacological therapies, including callosotomy (79).

#### Corpus Callosotomy

Corpus callosotomy is particularly effective in the treatment of atonic seizures and drop attacks, and has been shown to be more effective than VNS in this setting (84). This finding has been supported by a meta-analysis that compared seizure outcomes in nine studies in which corpus callosotomy was used to treat LGS with 17 studies in which VNS was used (85). Atonic seizure responder rate (>50% reduction) was significantly higher for corpus callosotomy versus VNS (80.0 vs. 54.1%; *p* < 0.05), as was the proportion of patients experiencing a >75% reduction in atonic seizure frequency (70.0 vs. 26.3%; *p* < 0.05). For all other seizure types and for total seizures, there were no statistically significant differences between VNS and corpus callosotomy (85).

In a study that compared the long-term (2 years) outcomes of patients with generalized epilepsy of the Lennox-Gastaut or Lennox-like type who underwent callosotomy (*n* = 24) or VNS (*n* = 20), both procedures were shown to be effective for atypical absences, generalized tonic–clonic seizures, and tonic seizures (86). Callosotomy was particularly effective in reducing the frequency of atonic seizures, whereas VNS was ineffective. In contrast, callosotomy was ineffective in reducing myoclonic seizures, whereas VNS was effective (86). A retrospective study conducted in Taiwan investigated the role of West syndrome in postcallosotomy seizure outcome in patients with LGS (87). Seventy-four LGS patients who underwent corpus callosotomy were followed up for more than 4 years, of whom 21 had a history of West syndrome and 53 did not. Overall, 16/21 (76.2%) patients who had a history of West syndrome and 29/53 (54.7%) patients who did not have history of West syndrome were responders (>50% seizure frequency reduction). There was no statistically significant difference in outcomes between the groups, indicating that a history of West syndrome does not appear to influence postcallosotomy outcomes in LGS (87). A prospective study conducted in 60 school-aged children with LGS compared the long-term effects of anterior corpus callosotomy versus AED treatment on seizure control, intelligence quotient, and quality of life (QoL) (88). Seizure freedom rates in the callosotomy versus AED treatment groups were 17.4 versus 2.9% at 1 year, 13.0 versus 5.9% at 2 years, and 8.7 versus 2.9% at 5 years (statistically significant at all timepoints). Significant differences in favor of callosotomy were also found for mean changes in intelligence quotient and overall QoL after 2 years, which were not related to postoperative outcomes of seizure control (88). A study conducted in 10 LGS patients demonstrated that VNS can be effective in controlling residual seizures following corpus callosotomy (89).

Based on available evidence and clinical experience, callosotomy appears to be particularly effective for treating drop attacks and it is therefore recommended that it should be considered in LGS patients for whom drop attacks are especially problematic. Callosotomy may be considered early in the clinical course of such patients, or when other treatment options have been tried, depending primarily on patient/parent/guardian choice. Following callosotomy, other treatments may also be used, if required, including VNS. There is currently no evidence for a disconnection syndrome in children with LGS who undergo callosotomy.

## General Principles of LGS Treatment

Lennox–Gastaut syndrome is a complex epileptic and developmental encephalopathy, with an extremely poor prognosis for long-term seizure control and cognitive outcome. Even with current and new pharmacological agents, seizure freedom is highly unlikely to be achieved (34). During all stages of life, management of LGS must carefully balance the need for treatment against side effects, with the patient’s overall QoL always being the primary focus (3), seeking optimal seizure control, rather than, necessarily, complete seizure freedom. Fluctuations in seizure frequency are likely to occur regardless of intervention, and the effect of an intervention should be assessed over an appropriate period of time (35). The aims of treatment may differ according to patient age and stage of disease, and this should be a primary consideration during patient re-evaluation and transition of care. In many patients, the main aim of LGS treatment is not necessarily to achieve seizure freedom, but to suppress or reduce the frequency of the more disabling seizure types (4). It is important to consider that a patient’s QoL may be impaired more by the side effects of treatment than by the seizures themselves (4) and clinicians should always be vigilant for adverse drug effects.

Treatment goals should be agreed with the parents/caregivers and, if possible, the patient before selecting a treatment plan (4). Assessments of QoL are more important in the long term than measurements of seizure outcome (4). Standardized measures of cognitive performance and behavior are also important over the long term (4). Treatment plans should be regularly reassessed, and agreement of the treatment plan should include a comprehensive and transparent explanation of the type, severity, and duration of AEs that might be expected to occur, how these should be managed, and the reasons for fast or slow titration/tapering off of AEDs. As for all types of epilepsy, polytherapy (AEDs and comorbidity-associated medications) should be rationalized and minimized whenever possible. The rationale/need for specific AEDs should be considered routinely as part of patient re-evaluation. In addition, clinicians should proactively ask the patient/parent/caregiver about AEs and not expect spontaneous reporting.

## Treatment Algorithms for LGS

The following recommendations and practical advice are the authors’ expert opinion, based on the available evidence and their clinical experience.

The full clinical features diagnostic of LGS may evolve with time, and this must consequently remain a consideration with regard to planning therapy in young children presenting with multiple seizure types. Since it may take time for patients to develop all the clinical and EEG features of LGS, it is recommended that—once all attempts to rule out other diagnoses have been undertaken—a patient presenting with symptoms suggestive of LGS should be treated as though they have LGS (as outlined in the Section “Newly Diagnosed Patients with LGS”) until their full clinical/EEG profile becomes clear.

### Newly Diagnosed Patients with LGS

**Figure 1:**
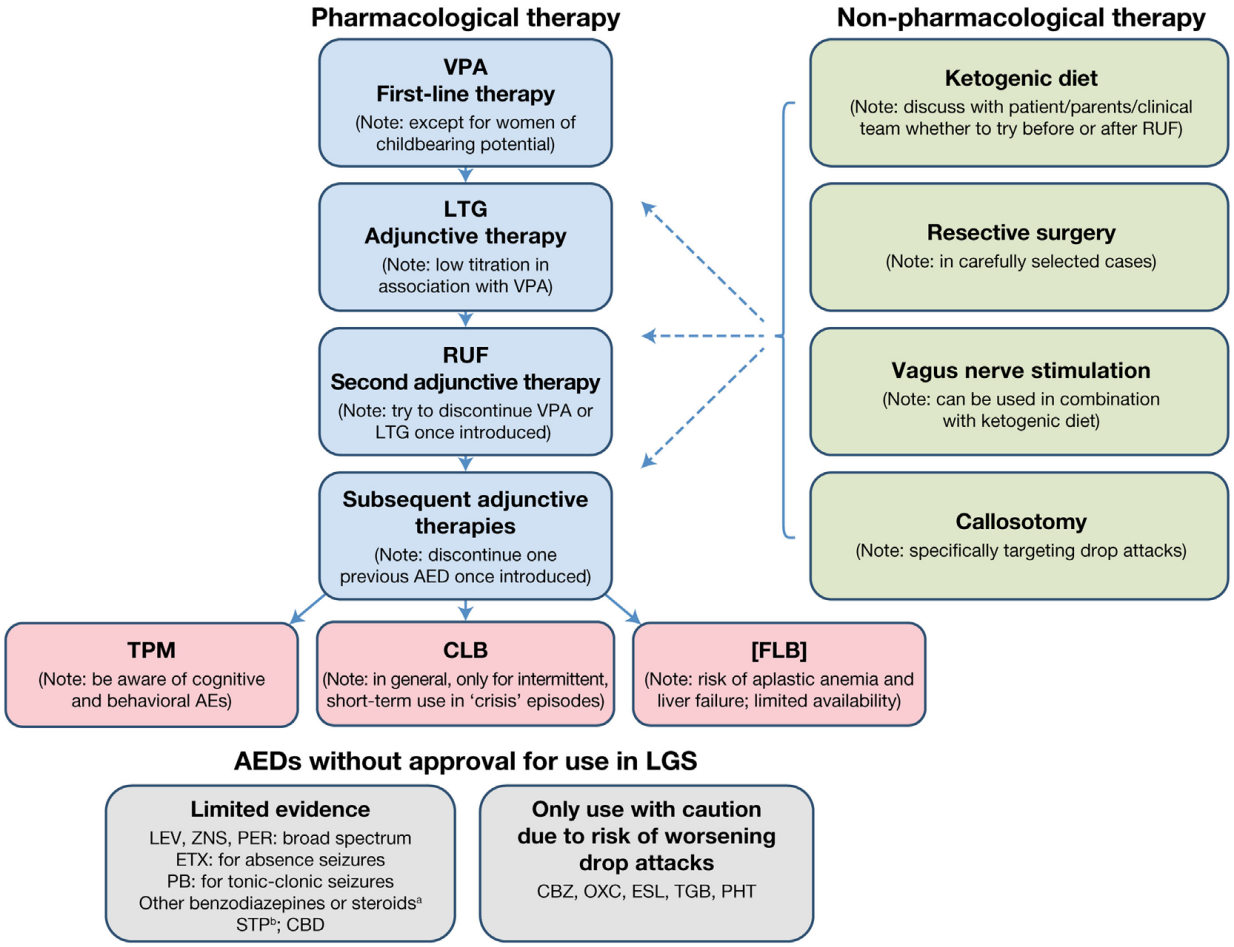
Treatment algorithm for a newly diagnosed patient with LGS. ^a^Not in combination and only for intermittent, short-term treatment of “crisis” episodes. ^b^In combination with VPA and/or CLB. AE, adverse event; AED, antiepileptic drug; CBD, cannabidiol; CBZ, carbamazepine; CLB, clobazam; ESL, eslicarbazepine acetate; ETX, ethosuximide; FLB, felbamate; LEV, levetiracetam; LGS, Lennox–Gastaut syndrome; LTG, lamotrigine; OXC, oxcarbazepine; PB, phenobarbital; PER, perampanel; PHT, phenytoin; RUF, rufinamide; STP, stiripentol; TGB, tiagabine; TPM, topiramate; VPA, sodium valproate; ZNS, zonisamide.

The treatment algorithm for a newly diagnosed patient with LGS is presented in Figure 1.

#### Patients with *De Novo* LGS

For a patient presenting with newly diagnosed *de novo* LGS, the recommended first-line treatment is VPA (Figure 1). If VPA therapy does not provide adequate seizure control, which is almost always the case, LTG should be added as the first adjunctive therapy. Since VPA inhibits LTG metabolism, a decreased LTG dose with slow titration should be used. If VPA plus LTG does not provide adequate seizure control, RUF should be initiated as adjunctive therapy. Once RUF has been initiated, attempts should be made to discontinue either VPA or LTG, and, if VPA is discontinued, the LTG dose should be increased. When considering adding an adjunctive therapy, every attempt should be made to discontinue one of the two previous AEDs once the new AED has been introduced, since there is no evidence for the effectiveness of more than two AEDs in combination, and the use of multiple AEDs unnecessarily raises the risk of side effects and/or drug–drug interactions.

If adequate seizure control is not achieved with the addition of RUF, the choice of the next adjunctive AED should be discussed with the patient/parent/caregiver/clinical team, based on the patient’s clinical profile and patient/parent/caregiver preference (e.g., available formulations). When discussing further AED options, the following points should be considered:

- Topiramate is licensed for LGS but has a greater potential for cognitive and behavioral adverse effects than other marketed AEDs.
- Consideration may be given to short-term treatment with CLB, temporarily increasing the AED load. There is a risk of tolerance, dependence, and cognitive/behavioral AEs. Consequently, CLB should preferably be used on an intermittent, short-term (3–5 days) basis when “crisis” episodes occur. Such crisis episodes include sustained absence seizures (duration >1 day), cluster seizures, and NCSE. This aside, where tolerance is not an issue, CLB may prove a useful adjunctive AED, particularly where drop attacks are troublesome.
- Felbamate is not licensed by the EMA due to associated risks of aplastic anemia and liver failure. It should only be used when other treatment options have failed and then only when the potential benefits of FLB treatment are thought to outweigh its risks. The use of FLB is likely to be country specific. If used, close monitoring (regular blood counts and liver function tests) is highly recommended.

Several AEDs that do not have a specific license for LGS may nevertheless be considered as adjunctive therapy options. LEV, ZNS, and PER have demonstrated some evidence of effectiveness in LGS. All are broad-spectrum in their modes of action and may therefore be useful in treating multiple seizure types. LEV particularly may be a useful adjunct as it has few interactions with other medications. Ethosuximide can be useful for the treatment of absence seizures, but should always be combined with an AED that is effective in treating generalized tonic–clonic seizures and tonic/atonic seizures, since it is not effective for these seizure types. Benzodiazepines other than CLB may be considered for intermittent, short-term use for “crisis” episodes (as recommended for CLB), but should not be used in combination with each other. Although there is no published evidence to support the efficacy of stiripentol (STP) in treating LGS, it can be used in combination with VPA and/or CLB. The usual approach is to add STP to VPA and subsequently add a small dose of CLB, if required. It should be noted that STP will increase VPA and CLB levels, so some dose adjustment will be required. There is also emerging evidence that cannabidiol may be effective and well tolerated as adjunctive therapy in LGS patients (see text footnote 11), although this requires confirmation in further trials and long-term safety studies. Carbamazepine, oxcarbazepine, eslicarbazepine acetate, tiagabine, and phenytoin should only be used with caution, due to the potential risk of aggravation of drop attacks with a myoclonic component (expert opinion).

The use of non-pharmacological treatment approaches should be considered alongside the use of AEDs and discussed from the outset as part of the patient’s treatment plan. An evaluation regarding resective surgery must be considered in all patients, particularly those with LGS with structural etiology who have lesions predominantly in one hemisphere or tuberous sclerosis. Some patients/parents/caregivers may choose to try KD therapy relatively early on in the patient’s treatment pathway. If this is the case, the KD can be tried if VPA plus LTG does not provide an adequate seizure response, before RUF is initiated. Alternatively, the KD may be introduced later, when multiple AEDs have been tried. Since a response to the KD is usually observed within 3 months (90), this therapeutic option can be explored relatively quickly. As callosotomy involves surgery, its use will largely depend on patient/parent/caregiver choice. Since it is effective for drop attacks, callosotomy may be considered as an early treatment option in patients for whom drop attacks are particularly problematic (e.g., if the patient suffers repeated injury from drop attacks, or is wheelchair-bound due to drop attack frequency). Re-evaluation with EEG and MRI is recommended before and after callosotomy to detect any changes resulting from the procedure (e.g., development of focal seizures). Although VNS is less invasive than callosotomy, it still involves a surgical procedure and its use will therefore largely depend on patient/parent/caregiver choice. It can take time for the effects of VNS to become apparent, with further improvement over time. The decision as to when VNS should be used will depend on a variety of factors, including age, time since LGS diagnosis, and whether the patient has been experiencing troublesome or intolerable AEs with AED treatment. VNS can be used in conjunction with AED therapy following callosotomy.

#### Patients with LGS That Has Evolved from Another Type of Epilepsy (e.g., West Syndrome)

Many patients will either have been treated with AED therapy to initially control seizures prior to LGS diagnosis, or will have developed LGS having progressed over time from another epilepsy syndrome, such as West syndrome. Most patients presenting with apparently generalized seizures will already be receiving VPA. If this is the case, then the treatment algorithm shown in Figure 1 can be followed as for a *de novo* LGS patient (i.e., adding LTG as the first adjunctive therapy if VPA does not provide adequate seizure control, etc). If the patient is already being treated with another first-line AED (typically LEV), then VPA therapy should be initiated and the other therapy tapered off and discontinued. Thereafter, the treatment algorithm is the same as for a *de novo* LGS patient (Figure 1).

If the patient is being treated with more than one AED and neither is VPA, then VPA therapy should be initiated and one of the previous AEDs tapered off and discontinued. If seizure control is inadequate after introducing VPA and the second AED is not LTG, then LTG should be initiated and the other non-VPA AED tapered off and discontinued. Thereafter, the treatment algorithm is the same as for a *de novo* LGS patient (Figure 1).

### Older Patients with Established LGS

It is recommended that patients with established LGS should undergo review by a neurologist on at least an annual basis, comprising a thorough reassessment of their diagnosis (in terms of epilepsy syndrome and etiology) and treatment plan. The diagnosis should be re-evaluated by repeating investigations conducted at initial diagnosis and/or conducting investigations that were previously not undertaken [EEG (including sleep-EEG, if possible), MRI, genetic testing], in order to confirm diagnosis and help elucidate etiology. Results of previous investigations should be reviewed alongside those of new investigations. Since the patient’s clinical and EEG features continue to evolve, a diagnosis other than LGS may become apparent and treatment should be adapted accordingly. Clinicians should always be alert to the possibility that the diagnosis may change and be vigilant to the possibility of treatable etiologies. Clinicians should also be aware that the “classic” EEG features (SSW complexes) may evolve and/or disappear later in the disease course (16, 91). Loss of these features does not necessarily mean that the patient no longer has LGS, but this possibility must be considered alongside reassessment of other clinical/EEG features. Genetic counseling should be offered, if appropriate. EEG should be repeated whenever there are any concerns over diagnosis, signs of deterioration, or suspected NCSE.

Existing treatment should be re-evaluated in terms of its effectiveness in controlling seizures, tolerability, impact on cognition/behavior, and impact on QoL, and adjusted if necessary. Patient notes should be reviewed and discussed with the caring physician. Every attempt should be made to rationalize polytherapy: ideally try to use no more than two AEDs in combination, except when CLB or another benzodiazepine is used acutely for a crisis episode (e.g., cluster seizures, prolonged absences, NCSE). The patient/parent/caregiver should be proactively asked about AEs and treatment adjusted accordingly. The patient’s cognitive ability and behavioral patterns should be reassessed regularly and treatment changed if it is suspected to be having a detrimental impact on the patient’s cognition/behavior. As for newly diagnosed patients, non-pharmacological treatment approaches should be considered alongside AED therapy when the patient’s treatment plan is reviewed. In adult patients, the withdrawal of treatment with a sodium channel blocker can be difficult, because generalized tonic–clonic seizures may appear. In such cases, slow tapering is recommended and the addition of CLB for a short period can be useful (92).

### Management of NCSE

Up to three-quarters of patients with LGS experience episodes of NCSE (4). Its clinical presentation can vary from a mild confusional state to coma (93). However, presentation in this population may be quite subtle; by definition NCSE is a change in behavior and EEG from baseline (94). The diminished responsiveness may be insidious in its onset, and therefore may be missed. A high index of suspicion is therefore required on the part of the physician, particularly if a previous baseline EEG is not available for comparison (94). Patients with LGS should be regularly assessed for the development of possible NCSE, including EEG, and, where possible, EEG results should be compared with a baseline recording (93). Expert advice may be required to confirm or refute a NCSE diagnosis.

Treatment for NCSE is less urgent than for convulsive status epilepticus^12^ and overtreatment should be avoided, since most patients do not require aggressive treatment. Patients should not be admitted to intensive care to induce an anesthetic treatment, since this could potentially be more harmful to the patient than the condition itself. NCSE should be treated with CLB and/or steroids (expert opinion). High-dose intravenous VPA (to achieve a target plasma level of >100–130 μg/mL) can also be effective (95). The goal of treatment is to return the EEG to its pre-NCSE baseline pattern, and treatment should be optimized with ongoing review. Patients with NCSE should be referred for specialist advice and/or EEG monitoring (see text footnote 12).

### Transition from Childhood to Adulthood

Transition from pediatric services into adult care is a difficult time for both patients and families, since educational provision ends and there are generally less resources available for adult than pediatric patients (3). Transition is particularly challenging for patients of intermediate age (16–17 years), since pediatric services are often reluctant to take on new patients of this age and keen to move existing patients to adult services (due to limited resources), while adult services will usually not take on patients aged <18 years. However, transition from pediatric to adult services also provides an important opportunity to carefully re-assess all aspects of patient care (3).

**Figure 2:**
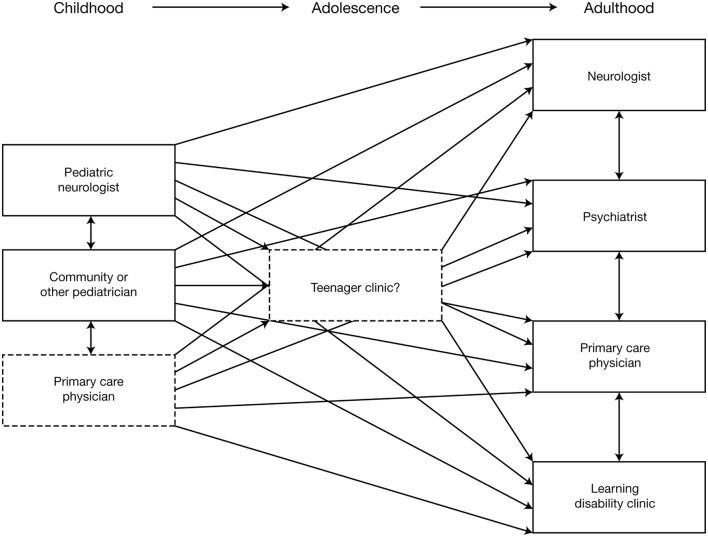
Schematic illustrating the increasing dispersion of care provision following transition from pediatric to adult services (3). Reproduced with permission from John Libbey Eurotext.

Etiology should be re-evaluated, using MRI and other investigations to exclude or detect specific etiologies that might affect treatment decisions; for example, tuberous sclerosis complex (96, 97). In addition to reviewing previous EEG reports, a “baseline” EEG at the time of transition is recommended. AED treatment should be re-evaluated to determine whether it is the most appropriate for the patient at that time and the use of polytherapy should be rationalized and minimized wherever possible. Similarly, patients should be reassessed to determine whether non-pharmacological therapy should considered (or re-evaluated, if already used). The multidisciplinary needs of the patient and their family/carers should be reassessed, in terms of the requirement for social care support, psychiatric support, and the provision of community or residential care. An important aspect of transitioning from pediatric to adult services is that provision of care becomes increasingly dispersed (Figure 2) (3). Specialist “transition clinics” or “teenager clinics” can help ease the transitional process (98, 99), and, for adolescent patients being treated with KD therapy, attendance at an adult epilepsy diet center can help ensure that an effective transition plan is in place (100). During the transition process, consideration should be given to the patient’s requirement for ongoing care by a physician with expertise in epilepsy (who may not necessarily be a neurologist). As previously stated, all patients should undergo at least annual review by a neurologist.

## Other Considerations for LGS Management

Lennox–Gastaut syndrome patients are impaired in their daily lives not only by a variety of seizure types that are often frequent and physically damaging (such as drop attacks), but also by a variety of serious comorbidities (3). Comorbidities that are particularly associated with LGS include cognitive and behavioral problems, physical disability, and sleep disturbances. Five years after LGS onset, 75–95% of patients have cognitive impairment (101, 102), and behavioral problems, such as hyperactivity, aggressiveness, and autistic traits, develop in approximately 50% (10). Mobility is often severely impacted by frequent seizures (particularly drop attacks), which are physically demanding and often result in injury (3). Patients often have to make use of protective equipment (such as a wheelchair, helmet, and/or faceguard) to minimize the physical effects of the seizures. Sometimes the use of protective equipment (e.g., remaining in a wheelchair to protect against injury from drop attacks) can itself impact the mobility of patients, feeding into a vicious circle that limits their ability to be physically active. In addition, the early development of dysphagia is strongly associated with poor long-term seizure prognosis (103), and affects patients’ ability to eat and take medication.

Patients with LGS suffer sleep cycle disruption due to the occurrence of seizures at night. Sleep deprivation and/or disruption affect the neurophysiological and neurochemical mechanisms important for the memory-learning process (104), and may also result in a wide range of behavioral, cognitive, and mood impairments, including hyperactivity, reduced school grades, and depression (105).

Careful management of comorbidities in LGS is a core aspect of care. Some AEDs may cause or exacerbate comorbidities (e.g., cognitive impairment, depression), and the choice of AED treatment must therefore take this possibility into consideration; for example, benzodiazepines used to treat sleep disorders may precipitate tonic seizures in LGS (106). Careful consideration must also be given to the potential for AED drug–drug interactions with medications used for the treatment of comorbidities.

As a result of the substantial burden of seizures and comorbidities, and the side effects of associated medications, the QoL of LGS patients is impaired on many levels (physical, mental, social) throughout their lives (3). The physical impact of LGS, and the preventative measures taken to minimize this impact, severely affect patients’ ability to participate in everyday activities, and school attendance is often disrupted. Cognitive and behavioral problems often require specific educational and care needs that will prevent mainstream school attendance (3). In addition, the aforementioned impact of nocturnal seizures on sleep can directly impair patients’ QoL (107). The effects of LGS on independence, ability to work, social participation, and personal relationships continue to severely impair patients’ QoL into adulthood (3). LGS also has a major impact on the QoL of parents/carers and families due to restriction of social life and relationship problems between partners and other family members; feelings of isolation, which can lead to depression; problems with childcare, which can further restrict social participation and opportunities for respite; physical exhaustion and/or disrupted sleep; anxiety about when seizures will occur, the future prospects of the individual with LGS, and the social stigma associated with the condition; as well as financial hardship resulting from forgoing career development in favor of caregiving (108).

Since LGS constitutes a major burden for patients and their caregivers/families, a multidisciplinary, individualized approach to care is required, which addresses each patient’s medical, educational, psychological, and social needs throughout the course of their life (109). The needs of the patient and their caregiver/family should ideally be re-evaluated annually, taking into account factors such as the patient’s physical health, their potential need for institutionalization (particularly in adulthood), and support for the caregiver/family.

## Areas for Future Research

At present, AEDs are *anti-seizure* medications and therefore treat the symptoms of epilepsy rather than its cause(s). Future research should focus on elucidating the natural history of LGS and whether appropriate treatment can have a beneficial impact on its disease course. This should not only include the effects of pharmacological treatment but also the role of surgical procedures such as callosotomy. Early control of seizures in LGS has been suggested to be associated with improved neurocognitive outcomes (2), but the relevance of this in *de novo* LGS, as well as the impact of seizure suppression in older children and adults, is less certain, and is an area that warrants closer study. Although attention is currently given to the potential detrimental effects of AEDs on cognition, research is required to elucidate whether other non-AED treatments can protect against cognitive impairment and/or improve cognition in LGS patients. In addition, research is needed to develop standardized tools for carers to routinely measure changes in patients’ cognitive performance and behavior over time. Elucidation as to whether there are specific genotypes or genetic mutations that determine a susceptibility to LGS is also needed, which could direct the development of diagnostic tools.

Given the problems associated with counting seizures in LGS (due to frequency of drop attacks, length of absence seizures, etc.), the predictive value of using alternative endpoints in clinical trials research should be assessed, such as the number of seizure-free days, rather than seizure frequency *per se*. Characterization of factors that predict response to treatment in LGS patients would also be valuable in the research and clinical practice settings. Other potential areas of future research include the relevance of steroid therapy in LGS, and whether it can improve cognition [since there is some evidence of beneficial cognitive effects of steroid therapy for infantile spasms (110)], and the long-term effects of cannabidiol treatment in LGS, in terms of AEs and the potential development of tolerance.

## Author Contributions

All authors (JC, SA, MF, PS, and AA) made substantial contributions to the conception of this article and the analysis and interpretation of the data it contains; were involved in drafting the article or revising it critically for important intellectual content; provided final approval of the version to be published; and agreed to be accountable for all aspects of the work in ensuring that questions related to the accuracy or integrity of any part of the work were appropriately investigated and resolved.

## Conflict of Interest Statement

JC has sat on advisory boards for Eisai, UCB, Shire, Zogenix, Vitaflo, and Nutricia, for which payment and honoraria have been paid to her department. She has participated as an investigator for Vitaflo, GW Pharmaceuticals, and Zogenix, with funds paid to her department. She has also received research funds from Vitaflo. She is supported by the National Institute for Health Research Biomedical Research Centre at Great Ormond Street Hospital for Children NHS Foundation Trust and University College London. SA has sat on advisory boards for Eisai, GSK, Novartis, Nutricia, Shire, Ultragenyx, and Zogenix. He has participated as an investigator for Advicenne Pharma, Eisai, UCB, Ultragenyx, and Zogenix, with funds paid to his hospital or his research lab. MF has participated as an investigator for UCB, GW Pharmaceuticals, Pfizer, and LivaNova, with funds paid to her department. She has received research funds from Eisai, UCB, LivaNova, and Esteve, with funds paid to her department. She has received speaker honoraria from Eisai, UCB, LivaNova, Bial, and Esteve. PS received honoraria from FB Health, Kolfarma s.r.l., UCB Pharma, and Eisai Inc., and research support from the Italian Ministry of Health and the Telethon Foundation. AA has received research grants from the European Commission, UCB Pharma, and CaixaBank, with funds paid to his department, and consulting fees from Eisai, GW, Shire, Takeda, Zogenix, and UCB Pharma.

## Footnotes

^1^Lamictal (lamotrigine) summary of product characteristics: http://www.ema.europa.eu/docs/en_GB/document_library/Referrals_document/Lamictal_30/WC500008824.pdf.

^2^Lamictal (lamotrigine) prescribing information: https://www.accessdata.fda.gov/drugsatfda_docs/label/2009/020241s037s038,020764s030s031lbl.pdf.

^3^Inovelon (rufinamide) summary of product characteristics: http://www.ema.europa.eu/docs/en_GB/document_library/EPAR_-_Product_Information/human/000660/WC500032937.pdf.

^4^Banzel (rufinamide) prescribing information: http://www.accessdata.fda.gov/drugsatfda_docs/label/2015/021911s012lbl.pdf.

^5^Topamax (topiramate) summary of product characteristics: http://www.ema.europa.eu/docs/en_GB/document_library/Referrals_document/Topamax_30/WC500018620.pdf.

^6^Topamax (topiramate) prescribing information: http://www.accessdata.fda.gov/drugsatfda_docs/label/2012/020844s041lbl.pdf.

^7^Frisium (clobazam) summary of product characteristics: http://www.medicines.org.uk/emc/medicine/8298/SPC/Frisium+Tablets+10+mg.

^8^Onfi (clobazam) prescribing information: http://www.accessdata.fda.gov/drugsatfda_docs/label/2011/202067s000lbl.pdf.

^9^Felbatol (felbamate) prescribing information: http://www.accessdata.fda.gov/drugsatfda_docs/label/2009/020189s022lbl.pdf.

^10^ClinicalTrials.gov: https://clinicaltrials.gov/.

^11^GW Pharmaceuticals Press Release, 27 June 2016: http://ir.gwpharm.com/releasedetail.cfm?ReleaseID=977116.

^12^National Institute of Health and Care Excellence (NICE) Clinical guideline 137: https://www.nice.org.uk/guidance/cg137/resources/epilepsies-diagnosis-and-management-35109515407813.

## Abbreviations

AE, adverse event; AED, antiepileptic drug; CLB, clobazam; EEG, electroencephalogram; EMA, European Medicines Agency; FLB, felbamate; KD, ketogenic diet; LEV, levetiracetam; LGS, Lennox–Gastaut syndrome; LTG, lamotrigine; MAE, myoclonic-astatic epilepsy; MRI, magnetic resonance imaging; NCSE, non-convulsive status epilepticus; PER, perampanel; RCT, randomized controlled trial; RUF, rufinamide; QoL, quality of life; SSW, slow spike-wave; STP, stiripentol; TPM, topiramate; VNS, vagus nerve stimulation; VPA, sodium valproate; ZNS, zonisamide.
